# What Characteristics Define Those Living Alone Across Stages of the Life Course?

**DOI:** 10.1093/geroni/igaf122.3020

**Published:** 2025-12-31

**Authors:** Aarohi Deshmane, Ross Andel

**Affiliations:** Arizona State University, Chandler, Arizona, United States; Arizona State University, Arizona State University, Arizona, United States

## Abstract

As mobile applications become increasingly used to support older adults’ mental health, these apps can provide useful insight about factors influencing health and wellbeing across the life course. We set out to investigate correlates of living alone in users of the mobile application Terrapino, launched in December 2022 and developed to help support cognitive health. The entire sample of Terrapino app users aged 18 years and older with complete information on sociodemographic, mental/physical health, and lifestyle factors (n = 8,395) were included and classified into age groups of 18-29 (n = 402), 30-44 (n = 1,128), 45-54 (n = 2,002), 55-64 (1,916), 65-75 (n = 2,078), and 75 + (n = 869). Overall, about equal proportion of individuals lived alone in the youngest (47%) and oldest (44%) age group, with ∼20-25% living alone in other age groups. However, men were significantly more likely to live alone early in adulthood (55% vs. 44%; χ2[1]=4.57, p=.033), whereas women were significantly more likely to live alone at ages 55-64, 65-74, and 75 + (χ2[1]=20.64, 127,43, 104.72, respectively, ps<.001). In fully adjusted logistic regression models, those living alone were less educated, engaged in more mental activity but also had poorer sleep, were more likely to smoke and be depressed, but were less likely to drink alcohol in high amounts (ps<.05). The differences, outside of sex differences, higher likelihood of depression and poor sleep in those living alone, started to emerge after age 44. Focusing on treating depression and improving sleep may serve as effective areas for intervention with those living alone at any stage of adulthood.

